# αA-Crystallin–Derived Mini-Chaperone Modulates Stability and Function of Cataract Causing αAG98R-Crystallin

**DOI:** 10.1371/journal.pone.0044077

**Published:** 2012-09-06

**Authors:** Murugesan Raju, Puttur Santhoshkumar, K. Krishna Sharma

**Affiliations:** 1 Department of Ophthalmology, University of Missouri–Columbia School of Medicine, Columbia, Missouri, United States of America; 2 Department of Biochemistry, University of Missouri–Columbia School of Medicine, Columbia, Missouri, United States of America; University of Durham, United Kingdom

## Abstract

**Background:**

A substitution mutation in human αA-crystallin (αAG98R) is associated with autosomal dominant cataract. The recombinant mutant αAG98R protein exhibits altered structure, substrate-dependent chaperone activity, impaired oligomer stability and aggregation on prolonged incubation at 37°C. Our previous studies have shown that αA-crystallin–derived mini-chaperone (DFVIFLDVKHFSPEDLTVK) functions like a molecular chaperone by suppressing the aggregation of denaturing proteins. The present study was undertaken to determine the effect of αA-crystallin–derived mini-chaperone on the stability and chaperone activity of αAG98R-crystallin.

**Methodology/Principal Findings:**

Recombinant αAG98R was incubated in presence and absence of mini-chaperone and analyzed by chromatographic and spectrometric methods. Transmission electron microscope was used to examine the effect of mini-chaperone on the aggregation propensity of mutant protein. Mini-chaperone containing photoactive benzoylphenylalanine was used to confirm the interaction of mini-chaperone with αAG98R. The rescuing of chaperone activity in mutantα-crystallin (αAG98R) by mini-chaperone was confirmed by chaperone assays. We found that the addition of the mini-chaperone during incubation of αAG98R protected the mutant crystallin from forming larger aggregates that precipitate with time. The mini-chaperone-stabilized αAG98R displayed chaperone activity comparable to that of wild-type αA-crystallin. The complexes formed between mini-αA–αAG98R complex and ADH were more stable than the complexes formed between αAG98R and ADH. Western-blotting and mass spectrometry confirmed the binding of mini-chaperone to mutant crystallin.

**Conclusion/Significance:**

These results demonstrate that mini-chaperone stabilizes the mutant αA-crystallin and modulates the chaperone activity of αAG98R. These findings aid in our understanding of how to design peptide chaperones that can be used to stabilize mutant αA-crystallins and preserve the chaperone function.

## Introduction

Alpha A-crystallin, a major structural protein of the vertebrate eye lens [1], belongs to the small heat shock protein (Hsp) family [2]–[4]. Like other members of this family, αA-crystallin exhibits chaperone-like activity [5]–[9]. The chaperone function of αA-crystallin prevents aggregation of unfolding proteins and is essential for maintaining transparency of the lens [6], [7]. In humans, the αA-crystallin gene is located on chromosome 21 and encodes a polypeptide of 173 residues [10]. Several point mutations have been reported in human αA-crystallin and these mutations cause structural changes in the protein and impair its chaperone activity. The loss of chaperone activity is considered one of the causes for the development of cataract [7], [11]–[13]. Congenital cataract is associated with R12C [14], R21L [15], R49C [16], R54C [17], and R116C [18] mutations, which occur at the conserved arginine residues. Pre-senile cataract is associated with a novel G98R mutation in αA-crystallin [19]. In the G98R mutation, a bulky basic amino acid, arginine, replaces the neutral glycine. Earlier studies of the recombinant G98R mutant protein revealed altered structure, substrate-dependent chaperone activity and impaired oligomer stability compared to wild-type recombinant αA-crystallin [20]–[22]

Generally, mutant proteins are prone to misfolding in the endoplasmic reticulum (ER) and subsequent aggregation. Paradoxically, some mutant proteins seem to fold efficiently in the ER but are subsequently misfolded at their target sites due to modification in their microenvironment. Functionally impaired mutant proteins or protein aggregates are generally rapidly degraded by the intracellular quality-control system [23] but some escape the quality-control mechanisms. Misfolded proteins are a hallmark of several pathological conditions including cataract. Several lines of evidence suggest that small molecular chaperones would be potential therapeutic molecules for diseases associated with misfolded proteins. Collectively called pharmacological chaperones, such molecules include native ligands, substrate analogues and small peptides [24], [25], which bind to mutant proteins and stabilize the mutant proteins to the extent that they function normally in vivo as well as in vitro.

We identified the major chaperone site in αA-crystallin and demonstrated that a 19 amino acid peptide (αA70-88, KFVIFLDVKHFSPEDLTVK), representing the chaperone site in the protein, functions like a molecular chaperone [26]. We have designated such a peptide as a “αA-mini-chaperone.” The amino acid sequence of this mini-chaperone is a highly conserved region among several small Hsps [27] and structure analysis shows that αA-mini-chaperone region aligns to the β3 and β4 region in the αA-crystallin. Our studies revealed that the ααA-mini-chaperone is effective in suppressing aggregation of H_2_O_2_-induced χ-crystallin [28] and denaturing substrate proteins ADH, citrate synthase, insulin and α-lactalbumin [26], [29], [30]. The mini-chaperone also inhibits amyloid fibril formation and its toxicity [31]. Because both β-sheet structure and hydrophobicity are necessary for maximal activity of the mini-chaperone, we concluded that direct interaction between the chaperone peptide and client protein is responsible for chaperone-like activity.

In this study, we examined the effect of αA-crystallin–derived mini-chaperone on the stability and function of the mutant αA-crystallin G98R. We show that mini-chaperone stabilizes the unstable mutant protein. Compared to the mutant protein, the mini-chaperone–stabilized αAG98R has a better capacity to chaperone denaturing protein. Our studies demonstrate specific interaction between the mini-chaperone and the mutant αA-crystallin. Using synthetic mini-chaperone harboring a benzoyl phenylalanine (Bpa) residue in place of a Phe we found that the mini-chaperone interacts at least at 1∶1 ratio with mutant αAG98R subunits and the stabilized protein has the chaperone activity comparable to that of the WT-αA-crystallin.

## Materials and Methods

### Proteins and peptides

Recombinant wild-type αA-crystallin and αAG98R mutants were expressed and purified as described earlier [21]. In brief, the full-length human αA-crystallin cDNA cloned into pET-23d (+) vector (Novagen, Madison, WI) was used as a template to generate the G98R mutation. Both mutant and wild-type proteins were expressed in *E. coli* BL21(DE3)pLysS cells (Invitrogen, Carlsbad, CA) and purified by column chromatography. The purity of the proteins was checked by SDS-PAGE and the molecular mass was determined by mass spectrometry. The concentration of the mutant and wild-type protein was estimated using Bio-Rad protein assay reagent. Mini-chaperone peptide (DFVIFLDVKHFSPEDLTVK), also called αA-mini-chaperone, and Pro-substituted mini-chaperone (DFVPFLDVKHFSPEDLTVK) were supplied by GenScript Corp. (Piscataway, NJ). Biotin-DFVIFLDVKH(Bpa)SPEDLTVK was supplied by Aapptec (Louisville, KY). The peptides used in the study were >95% pure as determined by high-performance liquid chromatography (HPLC) and mass spectrometry (MS). Alcohol dehydrogenase (ADH) was obtained from Biozyme, (San Diego, CA). All other chemicals were of the highest grade commercially available.

### Aggregation and multi-angle light scattering studies

αAG98R or wild-type αA-crystallin (75 µg) were incubated in the presence and absence of αA-crystallin–derived mini-chaperone (10 µg) for 1 hr in 100 µl PO4 buffer at 43°C, the temperature at which αAG98R readily aggregates [21]. Samples were injected on to a TSK G5000PW_XL_ (Tosoh Bioscience, Montgomeryville, PA) size-exclusion column equilibrated with 50 mM sodium phosphate buffer containing 150 mM NaCl (pH 7.2). The flow rate was set to 0.75 ml/min. The column was attached to a HPLC system connected with UV and refractive index detectors and coupled to a static multi-angle laser light scattering (DAWN-EOS) and dynamic quasi-elastic light scattering detectors (Wyatt Technology, Santa Barbara, CA). The molar mass (Mw), hydrodynamic radius (Rh) and polydispersity index (PDI) were determined using ASTRA (5.3.2) software developed by Wyatt Technology.

### Fluorescence spectroscopy

For measurement of intrinsic Trp fluorescence, protein samples (200 µg) were diluted in 1 ml of PO4 buffer (50 mM, pH 7.2, containing 150 mM NaCl) in the absence and the presence of mini-chaperone (10 µg). The sample was excited at 295 nm (slit width 5 nm) and the emission was recorded at 300–400 nm range (slit width 5 nm). The relative surface hydrophobicity of wild-type αA-crystallin and αAG98R proteins was measured using bis-ANS. Bis-ANS (1 mM) solution, 10 µl, was added to 0.2 mg protein in 1 ml buffer (50 mM phosphate buffer containing 150 mM NaCl, pH 7.2) in the absence and in the presence of mini-chaperone (10 µg). The samples were excited at 385 nm and the emission spectra were recorded between 400–600 nm using a Jasco spectrofluorimeter FP-750.

### Effect of αA-crystallin-derived mini-chaperone on chaperone activity measurements

The chaperone-like activity of wild-type αA-crystallin and αAG98R proteins was determined in the presence and absence of mini-chaperone using denaturing ADH as the aggregating substrate. Aggregation assay was performed in 1 ml 50 mM phosphate buffer containing 150 mM NaCl and 100 mM EDTA at 43°C. The chaperone activity of Biotin-DFVIFLDVKH(Bpa)SPEDLTVK as well as a Pro-substituted mini-chaperone (DFVPFLDVKHFSPEDLTVK) was also measured by ADH aggregation assay. The extent of aggregation was estimated by monitoring the light scattering at 360 nm using a Shimadzu UV-VIS spectrophotometer equipped with a temperature-controlled multi-cell transporter.

### Circular dichroism measurements

Circular dichroism (CD) spectropolarimeter, J815 (Jasco, Easton, MD), equipped with a temperature control system, was employed to record CD spectra. Far-UV CD measurements were carried out over the wavelength range of 190 to 250 nm with bandwidth 0.5 nm, scan speed 10 nm/min using 0.1-cm path length cuvettes. Protein samples were prepared in 10 mM sodium phosphate buffer (pH 7.2). Spectra are the average of five scans. Buffer signal was subtracted prior to reporting the data. Thermal denaturation data were collected from 25°C to 45°C, with protein concentration of 100 µg/400 µl. Thermal denaturation experiments were performed with a heating rate of 1°C/min, and CD signals at 218 nm were used to determine transition midpoints. Near-UV CD spectra were recorded using a protein sample of 1.5 mg/ml in the buffer used for far-UV studies.

### Confirmation of αA-crystallin–derived mini-chaperone binding to αAG98R protein

The interaction between αA-crystallin–derived mini-chaperone and αAG98R-crystallin was studied using benzoyl phenylalanine-substituted αA-mini-chaperone. Phenylalanine corresponding to Phe-80 in wild-type αA-crystallin was substituted in αA-mini-chaperone with benzoyl-phenylalanine (Bpa). αA-Mini-chaperone also contained a biotin moiety at the N-terminus, creating a biotinyl αA-mini-chaperone-Bpa peptide. Biotinyl-αA-mini-chaperone-Bpa peptide (200 µg) was incubated with 200 µg of αAG98R crystallin in buffer, pH 7.2, at 37°C for 1 hr. Subsequently, the sample was filtered in a Microcon 10 kDa cut of filter (Millipore, Bedford, MA) to remove free peptides. The sample was photolyzed for 30 min, at 4°C, using a UV lamp (365 nm) held at a distance of 7 cm from the sample. The photolyzed sample was desalted using C18 zip tip spin columns (Thermo Fisher Scientific, Rockford, IL), as per the manufacturer's protocol, and the bound protein was eluted in 70% acetonitrile. The binding of αA-mini-chaperone to αAG98R was confirmed by MALDI-TOF/TOF mass spectrometry. The UV-photolyzed sample was subjected to SDS-PAGE and western blot analysis using antibody against biotin.

### Electron microscopy

To examine the aggregation of G98R mutant protein (100 µg) in the absence and presence of αA-crystallin–derived mini-chaperone (10 µg), the purified protein was incubated at 37°C or 40°C in 7.2 pH phosphate buffer and the samples were analyzed by transmission electron microscopy (TEM). Aliquots of 5 µl were withdrawn at different time intervals (0 min, 10 min, 30 min) and placed on carbon-coated, 200 mesh copper grids and left for 1 min. The excess solution was wicked away with a filter paper. The proteins on the grid were stained with 5 µl of freshly prepared 5% uranyl acetate solution for 10 min. This solution was then wicked off, and the grid was air-dried and then examined using a JEOL 1400 TEM (120 kV). The images were captured on a digital camera with 20,000 magnification and imaging software from Gatan Digital Micrograph (Gatan, Inc., Warrendale, PA). The protein samples incubated at 37°C in presence and absence of mini-chaperone for 8 hrs and processed as above was also examined by TEM.

## Results

Recombinant crystallin proteins were expressed and isolated according to the procedure described earlier [21]. SDS–PAGE analysis confirmed that both wild-type and mutant forms of recombinant αA-crystallins were as pure (>98%) as the proteins used in earlier studies [21], [22]. Size-exclusion chromatographic profile of the mutant protein gave an elution profile with an oligomer peak and a peak of dissociated subunits, indicating that the mutant protein has an unstable oligomeric assembly, as described earlier [22]. On the other hand, the wild-type αA-crystallin eluted from the same column as a single peak with the expected elution time for αA-crystallin oligomer. Incubation of αAG98R at 37°C led to gradual aggregation over a period of time, whereas incubation at 43°C resulted in rapid aggregation of the mutant protein, as we reported earlier [21].

### αA-Crystallin–derived mini-chaperone increases the recovery of soluble αAG98R

**Figure 1:**
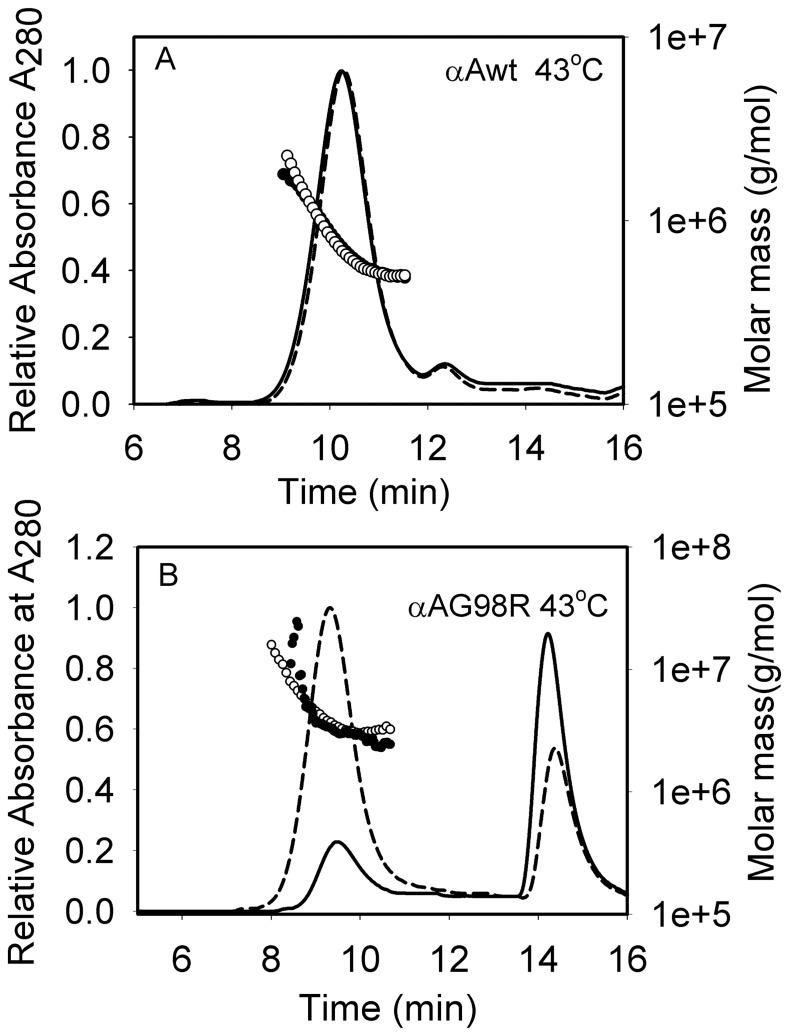
Molar mass distribution of αAG98R and wild-type αA-crystallin in the absence and presence of αA-crystallin–derived mini-chaperone. A, αA-crystallin wild-type (75 µg, solid line, filled circle); αA-crystallin wild-type (75 µg) in presence of 10 µg αA-mini-chaperone (broken line, open circle). Samples were incubated at 43°C for 30 min in PO4 buffer, pH 7.2, and were injected into a TSK-5000PW_XL_ (7.6 mm×30 cm) gel filtration column connected to a multi-angle light scattering instrument and the data was analyzed as described under methods. B, αAG98R-crystallin, 75 µg (solid line, filled circle) and αAG98R-crystallin (75 µg) in presence of 10 µg of mini-αA (broken line, open circle). The representative profile shows that αA-mini-chaperone suppresses the dissociation of subunits from αAG98R and increases the recovery of the mutant protein after incubation at 43°C and chromatography.

Following purification ofαAG98R, we examined the ability of αA-mini-chaperone to stabilize the mutant protein. We know from previous studies thatαA-mini-chaperone suppresses the aggregation and precipitation of denaturing proteins [26], [29], [30]. The purified αAG98R (75 µg), which aggregates and precipitates on incubation at 37–45°C [21] was incubated in the presence and absence of αA-mini-chaperone (10 µg) at 43°C for 1 hr. The samples were centrifuged to remove any precipitate formed during incubation, and the supernatant was analyzed by TSKG5000 PW_XL_ gel filtration column connected to a multi-angle laser light scattering (DAWN-EOS) and dynamic quasi-elastic light scattering detectors. The elution profile showed two peaks (Figure 1B). The first peak corresponded to the oligomeric form of αAG98R, whereas the second peak represented dissociated subunits of the mutant protein. In the absence of mini-chaperone, only 7.1 µg (9.5%) of the mutant protein was recovered, whereas 59.2 µg (79%) of the mutant protein was recovered when the incubation was carried out with αA-mini-chaperone. The monomeric peak also decreased in the presence of αA-mini-chaperone. The binding of αA-mini-chaperone to αAG98R was confirmed by HPLC analysis of the protein peak eluting between 8.5–11 min from the TSKG5000PW_XL_ column (the data are shown in Figure S1). Both αAG98R and αA-mini-chaperone were present in the protein peak, indicating that the peptide chaperone was in complex with αAG98R during gel filtration analysis. The average molar mass ofαAG98R oligomer (non-aggregated) recovered in the absence of αA-crystallin–derived mini-chaperone was 2.3×10^6^, whereas in the presence of the mini-chaperone, the average molar mass of the stabilized αAG98R was 2.8×10^6^, indicating that the αA-mini-chaperone prevented the dissociation of αAG98R protein and that the slightly increased molar mass might be due to binding of αA-mini-chaperone (Figure 1B). The hydrodynamic radius (Rh) of the stabilized αAG98R increased from 15.3 nm to 16.4 nm, consistent with increase in molar mass. Under similar experimental conditions, wild-type αA-crystallin oligomeric size and molar mass did not change upon incubation at 43°C (Figure 1A) and the mini-chaperone did not interact with wild-type αA-crystallin. This was confirmed by HPLC analysis of wild-type protein oligomer incubated with αA-mini-chaperone and isolated by gel filtration (Figure S1).

### Stabilization of recombinant αAG98R by αA-crystallin–derived mini-chaperone

**Figure 2:**
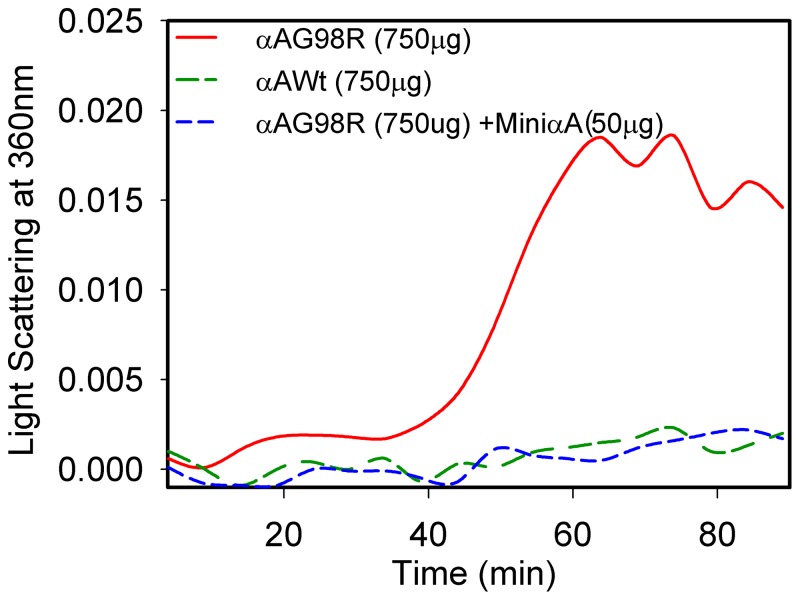
Thermal behavior of the wild-type αA-crystallin and αAG98R incubated with and without mini-chaperone. The protein samples (750 µg) were incubated in 1 ml PO4 buffer, pH 7.2 at 43°C and light scattering was continually monitored at 360 nm for 90 min in a spectrophotometer. The results show that mini-chaperone prevents the light scattering by aggregates formed by αAG98R incubated at 43°C. The figure is representative of 3 independent experiments.

To investigate the thermal behavior of mutant αAG98R protein and the effect of αA-mini-chaperone on αAG98R stability, we incubated the mutant protein (750 µg) at 43°C in the presence and absence of mini-chaperone, in a 1.7∶1 (mol/mol) ratio. Light scattering was monitored at 360 nm for 90 min using a spectrophotometer. As shown in Figure 2, αAG98R begins to form light scattering aggregates in 40 min. It is well known that under similar conditions, the wild-type αA-crystallin does not form light scattering aggregates. The chaperone peptide DFVIFLDVKHFSPEDLTVK, is known to suppress aggregation of proteins denatured by heat [26], chemicals [30] and oxidation [28], completely suppressed αAG98R aggregation (Figure 2). Because αAG98R is a structurally perturbed protein [20], [21], we hypothesize that the mini-chaperone interacted with mutant αAG98R and prevented aggregation and light scattering. Under similar conditions, incubation of αAG98R with a Pro substituted mini-chaperone (DFVPFLDVKHFSPEDLTVK), which has no chaperone activity (Figure S2), failed to suppress precipitation of the mutant protein (data not shown). The aggregation and precipitation of αAG98R also occurred at 37°C but at a slower rate. It took ∼8 hrs to see light scattering by αAG98R at 37°C and addition of αA-mini-chaperone completely suppresses light scattering (data not shown). In a separate experiment, when different amounts (1–30 µM) of mini-αA-crystallin was used with 10 µM of αAG98R in incubations at 43°C for 30 min, there was an increased recovery of mutant protein in soluble form from the reaction mixtures that contained higher amounts of chaperone peptide. Nearly 80% of αAG98R was recovered when the incubation was carried out with 1∶2 ratio (mol/mol) of αAG98R to mini-αA-. Further analysis of the αAG98R recovery data gave a Kd value 5.1 µM indicating the peptides high affinity to mutant protein.

### Morphology of αAG98R aggregates and stabilization by αA-crystallin–derived mini-chaperone

**Figure 3:**
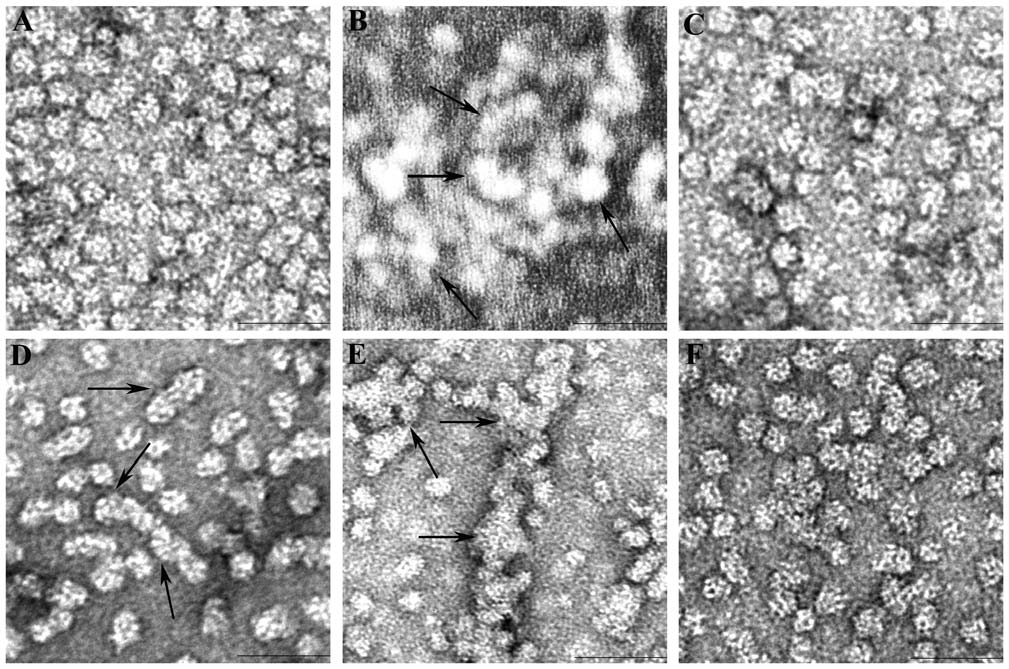
TEM micrographs of αAG98R mutant protein in presence and absence of αA-mini-chaperone. A, αAG98R – at 0 min at room temperature ; B, αAG98R incubated at 37°C for 8 hrs; C, αAG98R co-incubated with αA-mini-chaperone at 37°C for 8 hrs; D, αAG98R incubated at 40°C for 10 min; E, αAG98R incubated at 40°C for 30 min and F, αAG98R co-incubated with αA-mini-chaperone at 40°C for 30 min. Bar in the graph = 50 nm. The samples were examined under TEM as described under methods. The figures shown are representative of experiments with 3 different preparations of αAG98R and mini-chaperone. The arrow marks show the formation of aggregates of oligomers during incubation at 37 and 40°C. TEM analysis of αAG98R incubated with αA-mini-chaperone shows that the mini-chaperone prevents aggregation of αAG98R oligomers.

We examined under TEM the αAG98R incubated at 40°C to observe the aggregation pattern over a 30 min period. During TEM visualization the αAG98R incubated at 40°C showed formation of smaller aggregates comprising 2–10 oligomers in 10 min (Figure 3D). With longer incubation, several oligomers came together to form larger aggregates, and after 10 min of incubation, the smaller aggregates coalesced to give an amorphous appearance in 30 min (Figure 3E). Further we also observed that the αAG98R oligomer size of <15 nm gradually increased to a larger asymmetric form (∼20 nm) in ∼10 minutes of incubation at 40°C. Further incubation of the mutant protein resulted in larger, irregularly shaped aggregates that precipitate. However, αAG98R incubated in the presence of αA-mini-chaperone, in a mutant-to-peptide chaperone ratio of 1∶ 0.9 (mol/mol), did not form clusters or amorphous aggregates of oligomers (Figure 3 F). Additionally, the size of αAG98R oligomer incubated with αA-mini-chaperone was slightly smaller than the mutant incubated alone for 10 min at 40°C. A similar pattern of aggregation and suppression of aggregation with mini-chaperone was also observed by TEM when the mutant protein was incubated at 37°C for 8 hrs (compare B and C in Figure 3).

### Structural changes in αAG98R in the presence of αA-crystallin–derived mini-chaperone

**Figure 4:**
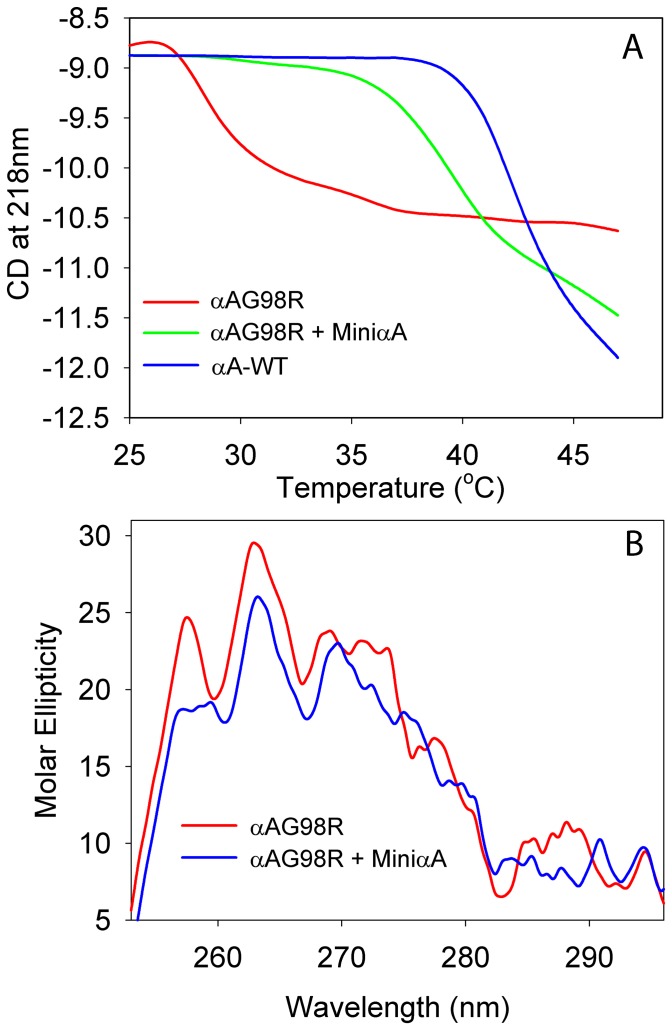
A, Far-UV CD analysis during thermal denaturation of αAG98R in the presence and absence of αA-mini-chaperone. Proteins, 100 µg, were prepared in 400 µl of10 mM phosphate buffer, pH 7.2, and the sample temperatures were slowly raised from 25 to 48°C in 2°C steps, equilibrated for 5 min at each temperature prior to far-UV CD measurement. Molar ellipticity changes at 218 nm were plotted to determine relative stability of samples. B, Near-UV CD spectra of αAG98R and αAG98R+αA-mini-chaperone. The spectra were recorded using a protein sample of 1.5 mg/ml. The profile shown is the average of 5 scans. The far-UV CD results show that the αA-mini-chaperone has the stabilizing effect on αAG98R.

The thermal behavior of αAG98R mutant in the presence and absence of αA-mini-chaperone was investigated at both near-UV and far-UV range, using a CD spectrometer equipped with a temperature controller. The temperatures of wild-type αA-crystallin and αAG98R mutant samples, from 25°C to 45°C, were raised slowly and negative ellipticity was recorded. The far-UV CD-spectra showed that wild-type αA-crystallin is very stable until 40°C, and at temperature above 40°C, the negative ellipticity at 218 nm increased with increasing sample temperature, indicating structural changes in the protein (Figure 4A). The mutant αA-crystallin began to show a significant increase in ellipticity above 27°C, and at temperatures above 40°C, the increases in ellipticity were moderated. Incubation αAG98R with αA-crystallin mini-chaperone stabilized the protein, as the negative ellipticity at 218 nm remained stable up until 35°C. Above 35°C, there was a gradual increase in 218 nm ellipticity, suggesting structural changes in the mutant protein occur at these temperatures even in presence of mini-chaperone. The near-UV CD spectrum of αAG98R–mini-chaperone was similar to that of wild-type protein in 272-260 nm region, whereas the spectrum in the 300-272 nm region showed minor changes suggestive of interactions between αAG98R and the mini-chaperone (Figure 4B) but the minimal nature of the interaction may be indicative of the interactions occurring away from the aromatic residues. This is supported by the absence of the peptide effect on intrinsic tryptophan fluorescence of αAG98R (Figure S3).

### Chaperone activity of αAG98R is stabilized after interaction with αA-crystallin–derived mini-chaperone

**Figure 5:**
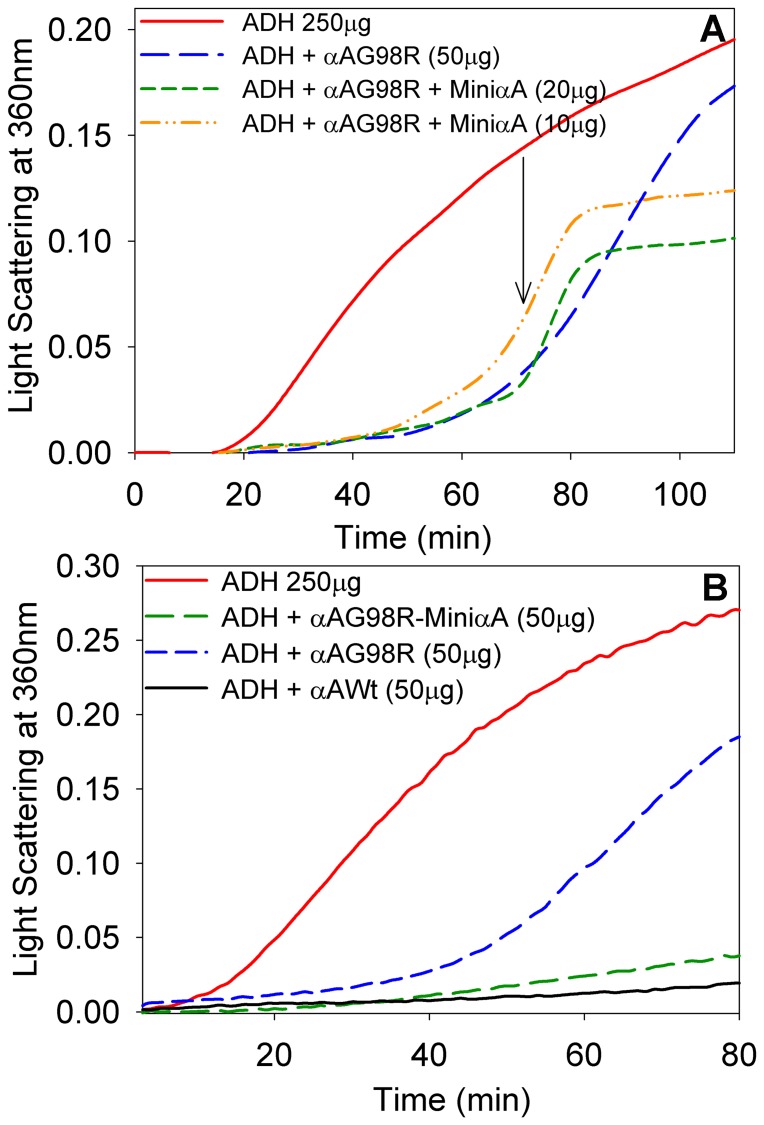
Effect of the addition of αA-crystallin–derived mini-chaperone on the chaperone-like activity of αAG98R against ADH aggregation. A. The aggregation of 250 mg of ADH at 43°C in 1 ml of phosphate buffer (50 mM, pH 7.2+0.15 M NaCl+10 mM EDTA) was measured in the presence and absence of αA-mini-chaperone. αA-mini-chaperone (10 µg or 20 µg) was added at 70 min after initiation of heat-induced aggregation (shown by arrow) and the assay was continued for 120 min. B, Comparison of chaperone activity of αAG98R, of αAG98R stabilized with αA-mini-chaperone and of wild-type αA-crystallin. The assays were performed as described under methods using 250 µg of ADH and 50 µg of crystallins. αAG98R stabilized with αA-mini-chaperone was obtained by mixing αAG98R with αA-mini-chaperone and isolating the complex by TSK5000PW_XL_ chromatography. The figures represent the typical data obtained multiple times with WT-αA-crystallin and stabilized αAG98R. The results show that the chaperone activity of αA-mini-chaperone stabilized αAG98R is comparable to that of wild-type αA-crystallin whereas the non-stabilized αAG98R has significantly reduced chaperone activity.

Earlier we reported that, compared to wild-type αA-crystallin, αAG98R mutant protein showed chaperone activity against denaturing ADH at 37°C during the early phase of the assay [21]. However, αAG98R chaperone activity diminished after 30 min of reaction at 43°C and precipitation of proteins was observed [21]. SDS-PAGE analysis of the precipitate revealed that αAG98R protein co-precipitated along with substrate ADH. We postulated that the precipitation was due to the unstable nature of ADH–αAG98R complex, and investigated whether αA-mini-chaperone would stabilize the complex. Similar to our earlier observation [21], αAG98R suppressed ADH aggregation during the early part of the 2 hr assay but the assay mixture started to scatter light after 60 min (Figure 5A). However, the addition of αA-crystallin–derived mini-chaperone to the reaction mixture of ADH+αAG98R significantly reduced the aggregation of denaturing protein (Figure 5A). It should be noted that the addition of αA-mini-chaperone did not solubilize the aggregates already formed. The suppression of αAG98R aggregation beyond the point of the addition of αA-mini-chaperone could be either due to the effect of αA-mini-chaperone itself or due to the stabilization of the ADH-αAG98R complex by the αA-mini-chaperone. To examine the latter possibility, αAG98R protein was incubated with αA-mini-chaperone at 37°C for 30 min, and the αAG98R–αA-mini-chaperone complex was isolated by gel filtration using a TSK G5000PW_XL_ column and the chaperone activity of the complex was determined employing ADH aggregation assay. The αAG98R treated with αA-mini-chaperone exhibited better chaperone activity than the untreated αAG98R (Figure 5B). Further, the chaperone activity of αA-mini-chaperone–stabilized mutant protein was comparable to that of wild-type αA-crystallin.

### Confirmation of αA-crystallin–derived mini-chaperone binding to αAG98R

**Figure 6:**
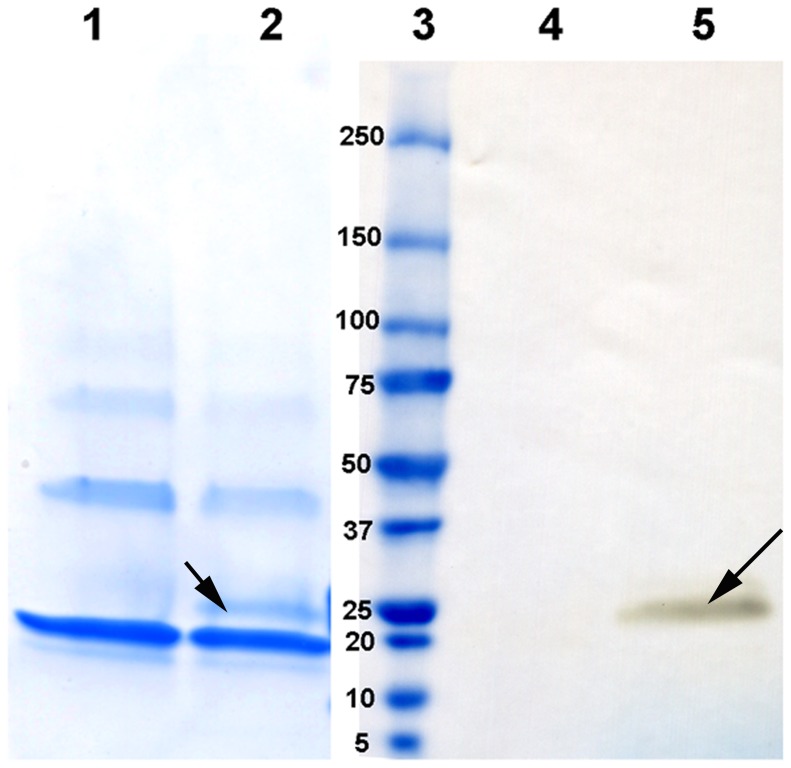
SDS-PAGE analysis of αAG98R cross-linked with biotinylated Bpa-mini-αA. Incubation, photolysis and SDS-PAGE (lanes 1–3), western blot (lanes 4 and 5) analysis were performed as described under methods. Lane 1, photolysed αAG98R; lane 2, αAG98R incubated with biotinyl-Bpa mini-αA and photolyzed; lane 3 mol wt markers. The arrow mark shows the αAG98R-biotinylated mini-chaperone complex formation during the photolysis. This band was absent in non-photolyzed samples (data not shown).Lanes 4 and 5 western blot of lanes 1 and 2 respectively. The stained band in lane 5 corresponds to the complex highlighted by arrow mark in lane 2 confirms the binding of a mini-chaperone to a subunit of αAG98R. The figure is representative of 3 independent experiments.

**Figure 7:**
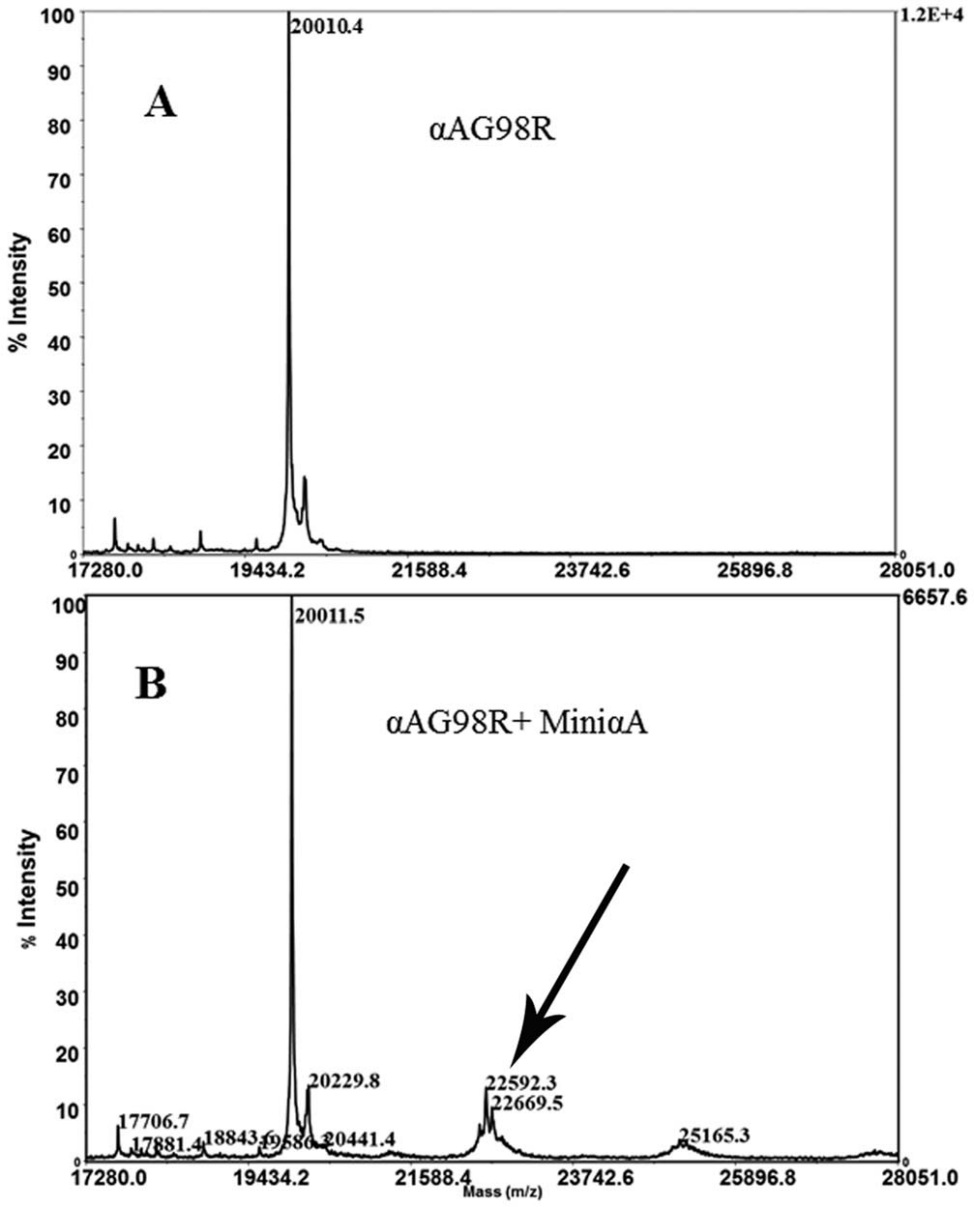
MALDI-TOF MS spectrum of αAG98R photolyzed after incubation with or without biotinyl-Bpa-mini-αA-crystallin. A, αAG98R after photolysis; B, αAG98R photolyzed after incubation with biotinyl-Bpa mini-αA. The αAG98R-Bpa mini-αA complex with m/z 22592 is indicated by an arrow. Mass spectrometric analysis confirms the 1∶1 interaction of mini-chaperone with αAG98R monomer. The figure is representative of 3 independent experiments.

We took advantage of photoaffinity labeling with Bpa, which was incorporated into the αA-mini-chaperone at one of the Phe sites, to elucidate the αA-mini-chaperone interaction with αAG98R. The biotin at the N-terminal of the peptide chaperone allowed the detection of the αA-mini-chaperone–G98R complex. Since biotin was attached at the N-terminus and the Bpa group was away from the critical Phe (corresponding to Phe 71 in αA-crystallin), the αA-mini-chaperone retained chaperone activity after these modifications. The photoaffinity labeling of αAG98R was performed using biotin-labeled Bpa–mini-αA and αAG98R. Excess Bpa-mini-chaperone was removed by filtration prior to photolysis. The photolyzed protein was analyzed by SDS-PAGE and western blot using avidin-horseradish peroxidase conjugate against biotin and mass spectrometry. Western blot of UV-irradiated mixture of αAG98R and Bpa-mini-αA separated by SDS-PAGE showed the presence of αAG98R–Bpa-mini-αA cross-linked protein band (Figures 6). The molecular weight of the biotin-containing protein band suggests that one peptide was incorporated into one subunit of αAG98R during photolysis. Image analysis of the stained gel showed that mini-chaperone–αAG98R had photo-crosslinked about 10% of αAG98R. MALDI TOF/TOF mass spectrometric profile of the photolyzed sample also showed that about 10% of αAG98R was bound with one biotin-Bpa-peptide (Figure 7B), whereas the unphotolyzed sample did not show binding of biotinyl-Bpa-mini-chaperone (Figure 7A).

## Discussion

The αAG98R mutation in αA-crystallin is associated with early-onset cataract [19]. We and others have shown that αAG98R protein has altered structure, stability and chaperone activity [20]–[22]. In vitro incubation of mutant protein at 37°C leads to the formation of soluble aggregates which, with time, coalesce and precipitate [21], [22]. Almost all of the mutant protein precipitates if incubation continues for several hours at 37°C or 40°C. This behavior is typical of many mutant forms of lens crystallins, such as χD mutants L5S, V75S and I90F [32], and αB mutants F27R [33] and D140N [34]. Aggregation and precipitation are hallmarks of cataract-causing crystallin mutations. The TEM studies show that αAG98R oligomers interact with one another to form clusters of 2 to 3 oligomers or linear structures composed of 3 to 8 oligomers in 10 min of incubation at 40°C (Figure 3). At 37°C, it takes 6–8 hrs to form such aggregates, whereas at the slightly higher temperature of 40°C, aggregation begins as early as 10 min (compare Figures 3B and 3D). With time, the aggregates coalesce to form irregular aggregates having several oligomers, as shown in Figure 3E. We do not yet know which residues on the surface of the oligomers are involved in oligomer dimerization or initial aggregation. Although each subunit in the oligomer has mutation and altered structure, only a few subunits in an oligomer may have a binding interface exposed to interact with another oligomer, since all the subunits are not equally positioned due to the irregular polydisperse nature of αAG98R oligomers [21]. Such a limitation of interaction sites would initially result in a linear arrangement or the formation of dimers and trimmers of the oligomer rather than the formation of an oligomer fully decorated with additional oligomers to form a cluster of several oligomers. With time, however, the aggregates of 2–10 oligomers would interact with one another to form amorphous aggregates, as shown in images of the 30 min sample at 40°C (Figure 3E). Because the mutant protein has an altered structure and increased hydrophobicity [20]–[22], we hypothesize that the G98R mutation exposes specific hydrophobic regions and these interact with other oligomers to form aggregates. Further studies are required to identify all of the exposed residues as a consequence of the G98R mutation. Alternately, it is possible that the increased chaperone property of the subunits in the mutant oligomer is responsible for recognizing another oligomer and this process could lead to the formation of aggregates of 2–10 oligomers. In support of this, it was shown earlier that αAG98R variant [21] and cataract-causing mutant of αA-crystallin R116C has enhanced affinity toward client proteins [35].

Earlier we discovered that a peptide representing the chaperone site of αA-crystallin is sufficient to suppress the aggregation of denaturing proteins. We showed that the peptide chaperone stabilizes the partially unfolded proteins [26]–[30] and prevents fibril formation by β-amyloid [31]. Since αAG98R has an altered structure, we investigated whether the αA-crystallin–derived mini-chaperone would prevent the mutant protein from precipitation during incubation. When αA-crystallin–derived mini-chaperone was incubated with αAG98R, we found that the αAG98R was stabilized and remained in solution (Figure 2) and the stabilized αAG98R can be isolated by chromatography (Figure 1 B). This observation was confirmed by TEM study (Figure 3), which showed that aggregation of αAG98R was prevented by the αA-mini-chaperone. We believe that the mini-chaperone interacts with the exposed hydrophobic regions of the mutant proteins and prevents these sites from binding to another oligomer to form aggregates that precipitate during the incubation at 37°C or 40°C. Further studies are required to confirm this since interaction of mini-chaperone with αAG98R did not result in significant change in hydrophobic probe Bis-ANS binding (Figure S3 A).

The addition of the mini-chaperone to the αAG98R sample prior to incubation at 43°C and chromatography by gel filtration increased by 8-fold the recovery of αAG98R in the soluble form (Figure 1B). However, an inactive form of mini-chaperone (DFVPFLDVKHFSPEDLTVK) did not prevent the precipitation of αAG98R, suggesting the chaperone activity of the peptide was responsible for maintaining the mutant protein in soluble form. The interaction of the αA-crystallin–derived mini-chaperone with αAG98R was confirmed by reversed-phase HPLC analysis of the αAG98R peak recovered following incubation of active mini-chaperone and mutant protein (Figure S1). Under similar experimental conditions, the wild-type αA-crystallin showed negligible interaction with mini-chaperone (Figure S1), suggesting that the conformational change in αAG98R perhaps acted as a chaperone sensor.

Peptides substituted with the photoactive amino acid Bpa have been used to confirm the interaction between ligand and receptor [36]–[38]. We substituted one of the three phenylalanines in αA-mini-chaperone with Bpa and biotinylated the N-terminal amino group to obtain biotin-DFVIFLDVKH(benzoylphenylalanine)SPEDLTVK. The chaperone peptide was active in suppressing the aggregation of heat-denatured ADH. When the biotinyl-Bpa-mini-chaperone–αAG98R incubation mixture was photolyzed and subjected to SDS-PAGE and western blot analysis, covalent association of αA-mini-chaperone with αAG98R subunits was observed (Figure 6A). The binding of biotinyl-Bpa-chaperone to αAG98R was also confirmed by MS analysis. The mass of the complex, 22592.3 m/z (Figure 6B) is equal to 1∶1 binding of αA-mini-chaperone and αAG98R subunit. We found that only about 10% of the Bpa-peptide was incorporated into αAG98R subunit. Bpa photocross linking efficiency is dependent on the duration of UV exposure, the affinity of the ligands and the orientation of the Bpa residue [37]. The low insertion of Bpa in our hands may in part be due to shorter photolysis time. We did not extend the photolysis time to minimize any UV-induced structural change in the protein that may influence the interaction of peptide with αAG98R. Further, it is unlikely that all subunits in the αAG98R oligomer interact with the mini-chaperone equally because of uneven exposure of hydrophobic regions to the surface in mutant crystallin.

Modulation of wild-type α-crystallin chaperone activity by small molecules such as ATP [39], glutathione [40], arginine and aminoguanidine [41], [42] has been previously reported. In those studies the modulator was used in 10- to 30-fold higher concentrations than the α-crystallin [39]–[42] and the conformational changes in α-crystallin in the presence of the modulator was considered to be responsible for the activity enhancement. Our study shows that 2-fold higher concentration of mini-chaperone is sufficient to stabilize the mutant αAG98R-crystallin in solution and the mini-chaperone stabilized crystallin has chaperone activity comparable to that of WT-αA-crystallin (Fig. 5B). We reported earlier that dithiothreitol (DTT) treatment of α-crystallin in the water-insoluble fraction of lens proteins restores some of the lost chaperone activity [43]. Oxidation of methionine in α-crystallin leads to loss of chaperone activity and this can be reversed by treatment with methionine sulfoxide reductase [44]. However, none of the studies carried out thus far attempted to rescue the chaperone activity in mutant forms of αA- or αB-crystallins. We have previously shown that αA-crystallin–derived mini-chaperone can suppress the aggregation of several proteins [26]–[30] and prevent fibril formation by β-amyloid [31]. This study is the first report on the stabilization of a mutant αA-crystallin by a mini-chaperone derived fromαA-crystallin. The rescuing of chaperone activity in αAG98R by αA-mini-chaperone can be compared to the interaction of a C-terminal peptide of p53 with inactive mutant forms of the same protein and restoration of its activity [45], [46]. The specific interaction between the αA-mini-chaperone and αAG98R subunit demonstrates the ability of the αA-mini-chaperone to act as a chaperone toward structurally perturbed αAG98R, akin to the mini-chaperone suppressing the aggregation of denaturing proteins [26], [30], [31].

In summary, we have shown that the αA-crystallin–derived mini-chaperone can suppress the aggregation of mutant parent protein. The increased stability of the mutant protein, coupled with only marginal increase in Rh in the presence of chaperone peptide suggests that the αA-mini-chaperone has the potential to become a therapeutic agent to stabilize the cataract-causing mutant forms of αA-crystallin. We propose that αA-crystallin–derived mini-chaperones or synthetic chaperones with mini-chaperone electrostatic surface would have the capacity to control the aggregation of crystallin-client protein complexes or conformationally challenged proteins. Further, peptide chaperones may serve as universal chaperones for controlling diseases involving protein aggregation.

## Supporting Information

Figure S1 **Elution profile of mini-αA, αAG98R-miniαA complex and αA-WT treated with αA-mini-chaperone from a C8 column.** 100 µg of αAG98R or WT-αA-crystallin and 10 µg of peptides were used in the study. Samples were passed through TSK5000pw column was used to separate αA-crystallin peak from the unbound peptides. The protein from the αA-crystallin peak was subsequently analyzed in a Vydac 208TP column (250 mm×4.6 mm) fitted to a Shimadzu HPLC system. Acetonitrile gradient (0–80%) over a period of 40 min was used to resolve the components. Eluent A was 0.1% trifluoroacetic acid in water and eluent B was acetonitrile. Detector was set at 220 nm and the flow rate 1 ml/min. A. Analysis of αA-mini-chaperone-αAG98R and αA-mini-chaperone. B. Analysis of αA-minichaperone and WT-αA-crystallin. The HPLC analysis of the fractions at α-crystallin elution region from gel filtration column shows the binding of αA-mini-chaperone to mutant protein but not to wild-type αA-crystallin. The figure is representative of 3 independent experiments.
(TIF)

Figure S2 **Chaperone assay in presence of either αA-mini-chaperone or αA-mini-chaperone with proline substitution.** The EDTA-induced aggregation of ADH assay was performed at 37°C as described under methods. In each experiment 250 µg of ADH was used. Curve 1, ADH alone; Curve 2, ADH+αA-mini-chaperone (pro) 50 µg; Curve 3, ADH+αA-min-chaperone, 50 µg. The results show that Pro-substitution abolishes the chaperone activity of mini-chaperone. The figure is representative of two independent experiments.
(TIF)

Figure S3 **Fluorescence studies of αAG98R in presence or absence of αA-mini-chaperone.** A, bis-ANS (1,1′-bi(4-anilino) naphthalene-5,5′-disulfonic acid) interaction with mutant protein before and after addition of αA-mini-chaperone was recorded as described under methods. The spectra shows minimal change in fluorescence after the peptide interaction with αAG98R. B, Intrinsic fluorescence spectra of αAG98R before and after addition αA-mini-chaperone. The data, representative of two independent experiments, shows minimum change in the bis-ANS binding or intrinsic tryptophan fluorescence in mutant protein following treatment with αA-mini-chaperone.
(TIF)
