# Supplementary figures and images for: αA-Crystallin–Derived Mini-Chaperone Modulates Stability and Function of Cataract Causing αAG98R-Crystallin
Source: PLoS One. 2012 Sep 6;7(9):e44077. doi: 10.1371/journal.pone.0044077 (PMC3435407; doi:10.1371/journal.pone.0044077)

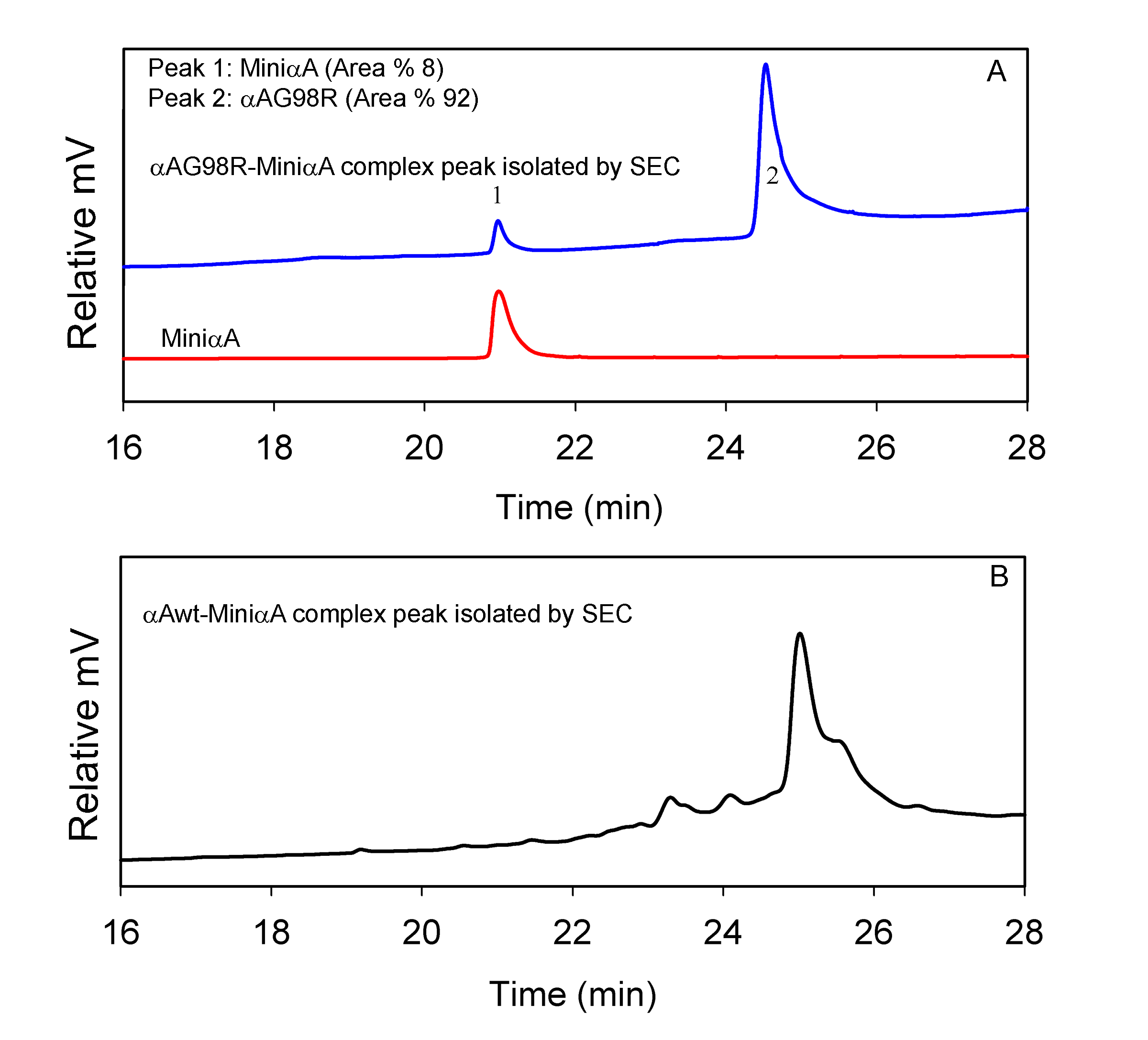

Supplement: Figure S1 — Elution profile of mini-αA, αAG98R-miniαA complex and αA-WT treated with αA-mini-chaperone from a C8 column. 100 µg of αAG98R or WT-αA-crystallin and 10 µg of peptides were used in the study. Samples were passed through TSK5000pw column was used to separate αA-crystallin peak from the unbound peptides. The protein from the αA-crystallin peak was subsequently analyzed in a Vydac 208TP column (250 mm×4.6 mm) fitted to a Shimadzu HPLC system. Acetonitrile gradient (0–80%) over a period of 40 min was used to resolve the components. Eluent A was 0.1% trifluoroacetic acid in water and eluent B was acetonitrile. Detector was set at 220 nm and the flow rate 1 ml/min. A. Analysis of αA-mini-chaperone-αAG98R and αA-mini-chaperone. B. Analysis of αA-minichaperone and WT-αA-crystallin. The HPLC analysis of the fractions at α-crystallin elution region from gel filtration column shows the binding of αA-mini-chaperone to mutant protein but not to wild-type αA-crystallin. The figure is representative of 3 independent experiments. (TIF) [file pone.0044077.s001.tif]

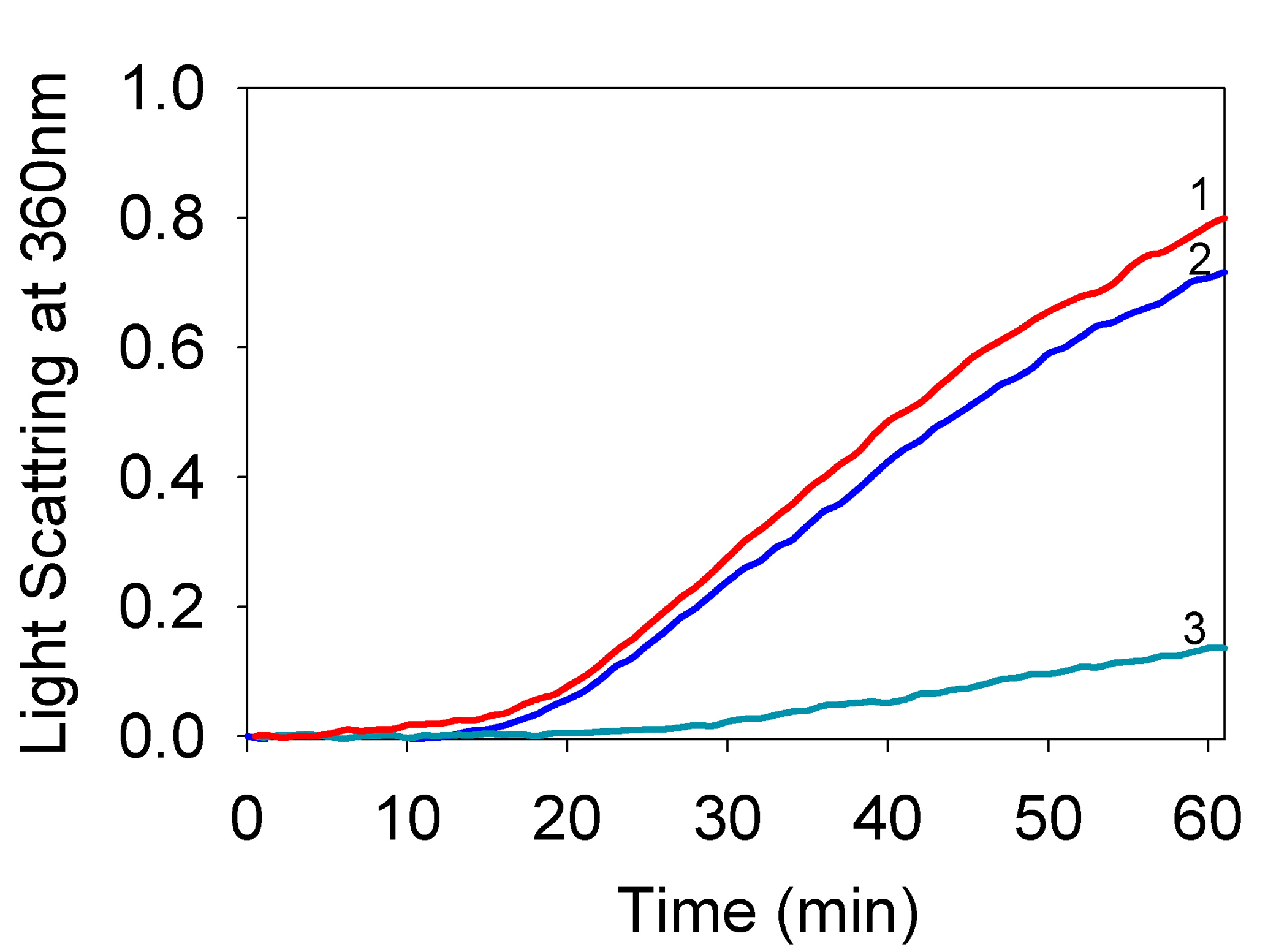

Supplement: Figure S2 — Chaperone assay in presence of either αA-mini-chaperone or αA-mini-chaperone with proline substitution. The EDTA-induced aggregation of ADH assay was performed at 37°C as described under methods. In each experiment 250 µg of ADH was used. Curve 1, ADH alone; Curve 2, ADH+αA-mini-chaperone (pro) 50 µg; Curve 3, ADH+αA-min-chaperone, 50 µg. The results show that Pro-substitution abolishes the chaperone activity of mini-chaperone. The figure is representative of two independent experiments. (TIF) [file pone.0044077.s002.tif]

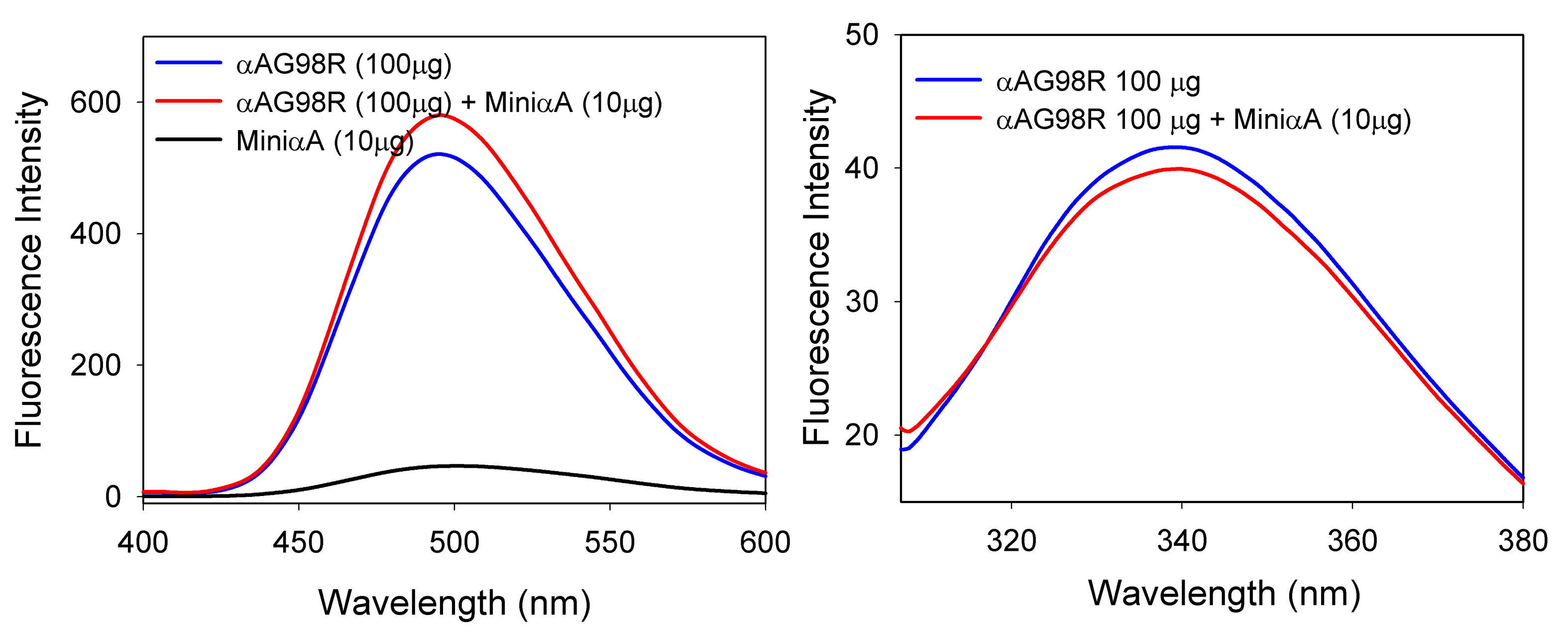

Supplement: Figure S3 — Fluorescence studies of αAG98R in presence or absence of αA-mini-chaperone. A, bis-ANS (1,1′-bi(4-anilino) naphthalene-5,5′-disulfonic acid) interaction with mutant protein before and after addition of αA-mini-chaperone was recorded as described under methods. The spectra shows minimal change in fluorescence after the peptide interaction with αAG98R. B, Intrinsic fluorescence spectra of αAG98R before and after addition αA-mini-chaperone. The data, representative of two independent experiments, shows minimum change in the bis-ANS binding or intrinsic tryptophan fluorescence in mutant protein following treatment with αA-mini-chaperone. (TIF) [file pone.0044077.s003.tif]
